# Response of peripheral arterial pulse wave velocity to acute exercise in patients after recent myocardial infarction and healthy controls

**DOI:** 10.1371/journal.pone.0219146

**Published:** 2019-07-09

**Authors:** Y. Trachsel, D. Herzig, T. Marcin, N. Zenger, M. Dysli, L. D. Trachsel, M. Wilhelm, P. Eser

**Affiliations:** 1 Preventive Cardiology & Sports Medicine, University Clinic for Cardiology, Inselspital, Bern University Hospital, University of Bern, Switzerland; 2 Institute for Biomechanics, ETH Zürich, Zürich, Switzerland; Linkoping University, SWEDEN

## Abstract

**Background:**

Many studies found increased central arterial stiffness and poor endothelial function in patients with coronary artery disease (CAD). Acute exercise has been shown to decrease peripheral pulse wave velocity (pPWV) in young healthy volunteers. We hypothesized the response to acute exercise to be diminished in CAD patients compared to healthy young (HY) and age-matched (HAM) controls.

**Methods:**

In 21 patients after recent myocardial infarction (CAD), 11 HAM and 10 HY pPWV was measured by applanation tonometry at the proximal femoral artery and the posterior tibial artery at rest and from 5 to 15 min after cessation of exhaustive exercise. Heart rate (HR) and blood pressure (BP) were monitored continuously. Resting central PWV (cPWV) was measured between the carotid and femoral arteries. Resting values and reponses to acute exercise were compared between the three groups and predictors for pPWV response were sought.

**Results:**

The response in pPWV to acute exercise seen in HY (lowering in pPWV by 17%) was absent in both CAD and HAM. Resting pPWV was not statistically different between the three groups, while cPWV was comparable in CAD and HAM but 17% lower in HY. Predictors for response in pPWV to exercise were age (Spearman r = 0.48), cPWV (r = 0.34) and response in diastolic BP (r = 0.32).

**Conclusion:**

The response in pPWV to acute exercise observed in HY was absent in CAD and HAM. In dilated peripheral arteries, PWV may reflect stiffness of passive vessel structures, which are likely to increase with age in healthy persons and CAD alike.

## Introduction

Exercise training in patients with coronary artery disease (CAD) has been shown to increase exercise capacity [1] and decrease cardiovascular and all-cause mortality [2]. Beneficial cardiovascular effects of exercise are manifold and include improved cardiac and pulmonary function, as well as a lowering of resting heart rate and systolic and diastolic blood pressure, the latter being caused by a reduced peripheral resistance [3]. Improved peripheral circulation after chronic exercise has been shown to be due to an increase in endothelial function, vasculogenesis and capillary density [3].

Due to the dominant effect of vasomotor tone in resting condition [4], dilation after occlusion or exercise offers a more reproducible state for measurements of vessel properties and the reaction to this challenge test provides valuable information of vascular reactivity. Dilation of peripheral large arteries as a response to acute exercise has been quantified by measuring artery diameter changes, flow changes or changes in pulse wave velocity (PWV). Several studies in heathy young cohorts have shown that there is a significant decrease in peripheral arterial PWV (pPVW) in the lower limbs immediately post-exercise which persists into the recovery period for approximately 30 min [5–13], while central PWV is not altered [14]. Previous studies in young healthy subjects and elderly cardiac patients have suggested that pPVW measured during hyperemia after occlusion may be a surrogate for flow-mediated dilatation (FMD, relative change in arterial diameter) following occlusion [15–18]. However, two studies that have directly compared post-occlusion pPWV measurements to FMD measured by ultrasound have found only partial agreement [17,19]. This implies that endothelial function is likely to play a role in post-exercise decrease of pPWV, but may not be the only important variable. Other important factors may be blood pressure [20] and vessel wall properties [21].

In CAD patients it has been shown that arterial stiffness is heightened [22] and endothelial function impaired compared to healthy populations [23]. Ankle-brachial PWV in combination with flow-mediated dilation has been shown to be useful for risk stratification of chronic CAD patients,[24] and exercise therapy has been shown to improve peripheral vascular function in these patients.[25] However, the responsiveness of peripheral arteries to peak exercise has not been measured in CAD patients before, and the need for studies assessing acute and chronic adaptations in populations with cardiovascular diseases has recently been pointed out.[26] We hypothesized that the response of peripheral arteries to acute exercise seen in young healthy people with pPWV decreasing acutely and transiently would be impeded in CAD patients. We further hypothesized that the response in pPWV to acute exercise would also be lower in CAD patients compared to healthy age-matched controls. Therefore, we measured pPWV at rest and acutely after exhaustive exercise in CAD patients with recent myocardial infarction, healthy aged-matched physically active men and healthy young men.

## Methods

### Subjects

Consecutive male CAD patients after recent myocardial infarction who were willing to enroll in a cardiac rehabilitation programme were recruited for the purpose of a related study (NCT02627586) at the University Clinic of Cardiology, University Hospital Bern, Switzerland between 1.9.2016 and 30.4.2018. Inclusion criteria were a first ST-segment elevation myocardial infarction or equivalent, with onset of symptoms of ischemia and treated by primary percutaneous coronary intervention within four weeks prior to study inclusion. Exclusion criteria were the inability to perform a maximal exercise test, a known chronic heart failure with left ventricular ejection fraction ≤ 45% before the acute index event, any medical intervention preventing a subject from performing exercise, or subjects with alcohol or drug abuse, or dementia.

Healthy young men (HY) between 20–35 years of age and healthy middle-aged men (HAM, matching the age distribution of the CAD population, between 40–75 years of age) were recruited from hospital staff and their friends or relatives. These subjects were recreationally moderately to very active (with a minimum of 60 min on at least 3 days/week). Exclusion criteria were smoking, hypertension (> 140/90 mmHg), a history of cardiovascular disease, diabetes and intake of regular medication. The study was approved by the Ethics Committee of the Canton Berne and all participants gave their written informed consent.

### Testing procedure

Subjects reported to the laboratory and changed into light sports gear. Height was measured using a wall-mounted stadiometer (KIRCHNER & WILHELM GmbH + Co. KG, Asperg, Germany), and weight was measured with a digital scale (InBody 720, Biospace Co., Seoul, Korea). Then they rested supine in a quiet, dimly lit room for 5 min. First, they had resting central and peripheral pulse wave velocity (PWV) measurements done. Then, still in supine position, they had resting heart rate (HR) and blood pressure (BP) recorded for 5 min while subjects were breathing at a paced rate of 0.25 Hz. After this, they mounted a stationary cycle ergometer and performed a cardiopulmonary exercise test with an individually determined ramp protocol (increasing by 10, 15, or 20 Watts/min) in order to reach exhaustion within 8–12 min. Oxygen consumption (VO_2_) was recorded continuously breath-by-breath in an open spirometric system (Jaeger Oxycon pro, ViaSys Healthcare GmbH) and peak VO_2_ was determined as the highest average of 8 breaths. After exhaustion, they performed a 2-min cool-down with light pedaling, after which they laid supine again for the 15 min post-exercise pPWV, BP and HR monitoring.

### Pulse wave velocity

Aortic (central) PWV (cPWV) was measured between the pulse sites of the carotid and femoral arteries by applanation tonometry (Sphygmocor, AtCor Medical, West Ryde, NSW, Australia). Distance between the location of the two pulse sites was measured using a caliper referenced against a measuring tape (100% of path length was used). Measurements were repeated if heart rate between both measurements differed by more the 5 bpm or if the standard deviation of the mean travelling time of the pulse waves (over the 10 s of the recording) exceeded 10% of the mean value.

Peripheral pulse wave velocity (pPWV) was measured between the right femoral artery and the right posterior tibial artery pre-exercise and continuously from 5 to 15 min post-exercise. Simultaneous recordings of the pressure waves of the femoral and the posterior tibial artery were obtained using an automated pressure transducer system (Complior SP, Artech Medical, Pantin, France). The distances between the measurement sites was again measured by caliper. The measurements taken post-exercise were obtained continuously and recorded by the median PWV of every 10 s.

### Heart rate and blood pressure

BP and HR were also measured pre-exercise and continuously from 5 to 15 min post-exercise. Baseline BP and HR mean values were calculated from a one-min period after a 3-min rest in supine position. BP and HR were measured by the Task Force Monitor (CNSystems Medizintechnik AG, Graz, Austria), which consists of a finger cuff (CNAP sensor) with pressure chambers and an infrared light sensor, an oscillometric upper arm blood pressure cuff, a four channel ECG and a corresponding software that enables beat-to-beat BP measurements on the finger artery.[27].

### Statistical analysis

Sample size calculation was based on previous studies who found statistically highly significant decreases in pPWV acutely after exercise in 12 and 25 healthy volunteers.[5,6] According to their results, 6 subjects are needed to find a decrease in pPWV at a one-sided alpha of 0.05 and a power of 80%. Therefore, we planned for 10 subjects in each of our healthy groups and 20 in our CAD group to accommodate for potentially missing data.

Statistical analysis was performed using the software R (Version 3.4.0, R Core Team, 2017). Baseline characteristics were compared between the three groups by ANOVA and between CAD and HAM/HY by t-test. Values at baseline and at 5 min post-exercise were compared between time points and between groups by mixed linear models (function lme of package nlme) with time point and group as fixed effects and subject as random intercept. Likewise, the same mixed linear models were also performed for data at each minute from 5 min to 14 min post-exercise.

The difference in peripheral PWV (delta pPWV) was calculated as peripheral PWV at 5 min post-exercise (or 6 or 7 min in 4 subjects who had no available PWV data at 5 min) minus peripheral PWV at baseline. Accordingly, delta systolic BP and delta diastolic BP were also calculated. A correlation matrix with Spearman correlation coefficients was composed with the following variables: Delta pPWV, age, VO_2_ peak, central PWV at baseline, peripheral PWV at baseline, mean BP, and difference in systolic BP from before to after exercise. Linear models were performed with delta PWV as dependent variable and as independent variables group, and parameters that had a correlation coefficient ≥ 0.3 with delta pPWV. Normal distribution of data was assessed by visual inspection of QQ-plots. A p-value of less than 0.05 was considered statistically significant.

## Results

Twenty-one CAD subjects, 11 HAM and 10 HY were recruited for the present study. Three CAD patients were excluded due to pPWV post-exercise measurements that only had a valid signal after more than 7 min post-exercise. One HAM participant was excluded because he turned out to have hypertension as assessed at the pre-exercise measurement. Baseline characteristics of the analysed 18 CAD, 10 HAM and 10 HY subjects are presented in Table 1.

**Table 1:** Comparison of baseline characteristics of the three groups.

Parameter	CAD	HAM	HY	p-value
Number of subjects	18	10**	10	
Age [yrs]	55.7 (7.3)	55.9 (9.5)	24.9 (3.0)***	0.000
Height [cm]	175.7 (8.5)	178.9 (6.9)	182.5 (6.0)*	0.085
BMI [kg/m^2^]	26.6 (3.7)	23.6 (1.8)*	24.2 (2.7)	0.029
*Resting parameters*				
Resting HR [bpm]	58.6 (8.3)	59.4 (7.7)	55.8 (6.5)	0.540
Resting systolic BP [mmHg]	111 (10)	120 (8)*	122 (12)**	0.013
Resting diastolic BP [mmHg]	73 (7)	80 (7)*	75 (5)	0.042
Resting mean BP [mmHg]	89 (7)	95 (10)	92 (6)	0.216
Resting pulse pressure [mmHg]	38 (6)	40 (7)	48 (11)**	0.014
Resting central PWV [m/s]	10.1 (1.3)	10.4 (2.0)	8.3 (0.7)**	0.003
Resting peripheral PWV [m/s]	9.5 (1.3)	9.6 (1.1)	9.1 (1.4)	0.577
*Peak parameters*				
Exercise test duration [min]	13.2 (2.4)	14.8 (2.3)	13.7 (2.8)	0.310
VO_2_ peak [ml/kg/min]	29.5 (5.6)	41.9 (6.5)***	54.1 (11.0)***	0.000
Peak HR [bpm]	143.2 (18.8)	173.2 (14.9)***	185.4 (10.4)***	0.000
HR reserve [bpm]	84.6 (19.9)	113.8 (15.8)***	129.6 (6.6)***	0.000
Peak systolic BP [mmHg]	124 (18)	135 (11)	141 (12)*	0.041

Shown are means (standard deviations). P-value are from ANOVAs with fixed factor group.

CAD, coronay artery disease group; HAM, healthy age-matched group; HY, healthy young group; BMI, body mass index; VO2 peak, peak oxygen uptake; HR, heart rate; BP, blood pressure; PWV, pulse wave velocity.

*, p≤0.05

**, p≤0.01

***, p≤0.001, all asterices refer to difference to reference group CAD by t-test

Disease characteristics and medication of the CAD group are given in Table 2. Mean beta-blocker dose was 27.8% of maximal dose. None of the CAD patients took vasodilators or Ca-antagonists.

**Table 2:** Baseline Characteristics and medication of the CAD group.

Parameter	18 CAD patients
*Disease severity*	
Ejection Fraction [%]	46.3 ± 12.8
3-vessel disease	9 (50%)
2-vessel disease	1 (4.5%)
1-vessel disease	8 (44.5%)
Number of stents	1.76 ± 1.30
*Cardiovascular Medication*	
Beta-blockers	18 (100)
Aspirin	18 (100)
Antithrombotics	18 (100)
Anticoagulants	2 (11)
ACE inhibitors/Sartans	17 (94.5)
Statins	18 (100)
Non-statins	2 (11)
Diuretics	4 (22)

Shown are means ± standard deviation or number of patients (percentage of all patients).

ACE, angiotensin-converting-enzyme.

**Fig 1:**
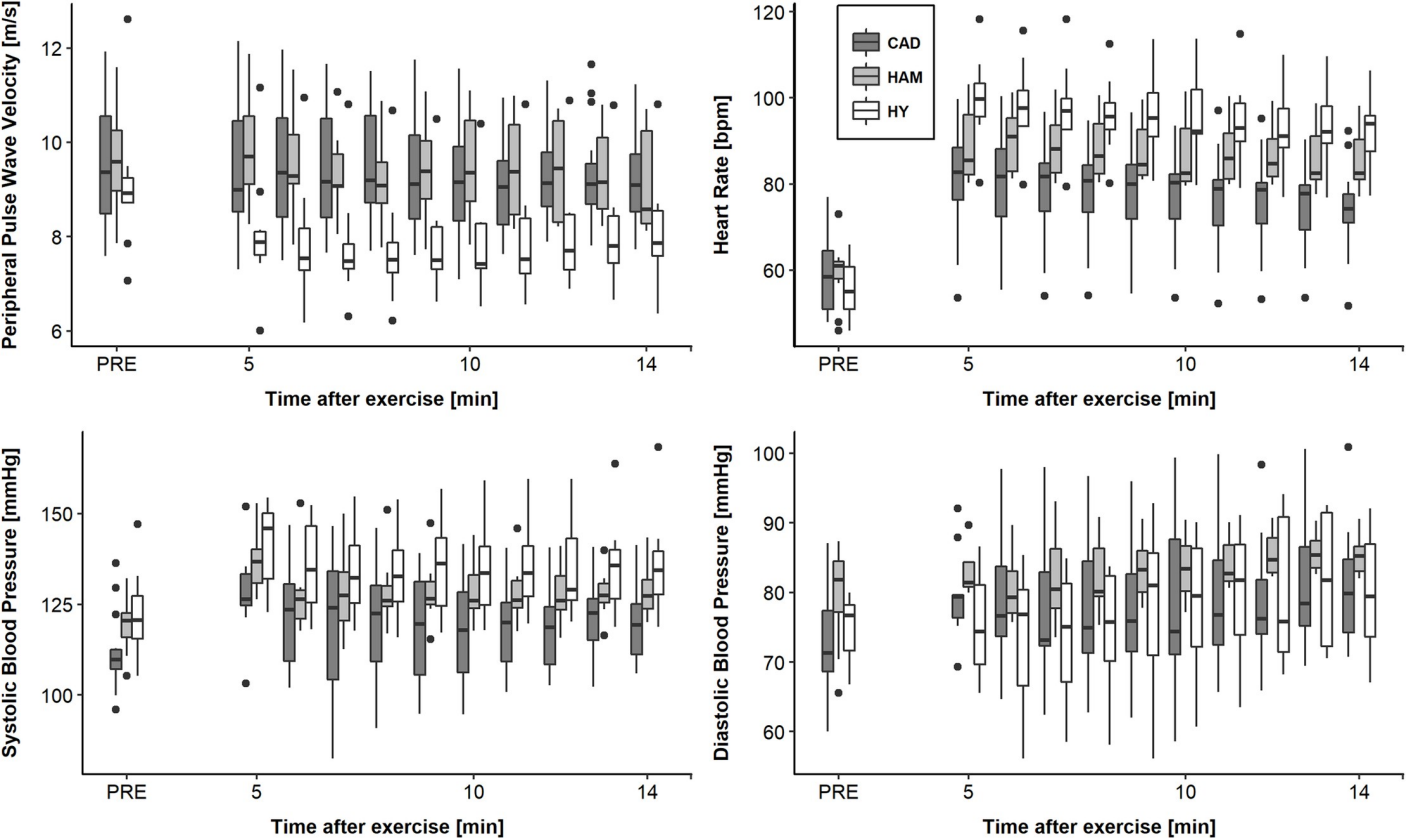
Box plots of data measured before (PRE) and from 5 to 14 min after the acute maximal exercise bout. Shown are peripheral pulse wave velocity (upper left panel), heart rate (upper right panel), systolic blood pressure (lower left panel) and diastolic blood pressure (lower right panel) of the CAD, HAM and HY groups. Boxes include the IQR, whiskers the outer quartiles without outliers, and outliers are defined as distance to median >1.5+IQR. CAD, coronary artery disease group; HAM, healthy age-matched group; HY, healthy young group; IQR, inter-quartile range.

PPWV was not different at 5 min post-exercise from baseline in the CAD and HAM group, while it decreased significantly by 1 m/s in the HY (p = 0.022, Table 3 and Fig 1). Between min 5 and min 14, pPWV decreased slightly and similarly in all groups (CAD group: 0.04 m/s per min, p = 0.022).

**Table 3:** Mixed linear models for pPWV and systolic as well as diastolic BP.

	Baseline to 5 min post-exercise			5 min to 14 min post exercise		
Fixed effects	Estimate	Std. Error	p-value	Estimate	Std. Error	p-value
*pPWV*						
CAD	9.52	0.30	0.000	9.66	0.32	0.000
HAM	0.12	0.50	0.810	0.46	0.52	0.388
HY	-0.44	0.50	0.387	-1.67	0.52	0.003
CAD x time interaction	-0.02	0.05	0.751	-0.04	0.02	0.022
HAM x time interaction	0.02	0.08	0.807	-0.02	0.03	0.382
HY x time interaction	-0.19	0.08	0.022	0.04	0.03	0.136
*Systolic BP*						
CAD	111.0	2.52	0.000	126.01	4.26	0.000
HAM	8.88	4.21	0.042	12.33	7.88	0.129
HY	11.44	4.21	0.010	16.86	7.73	0.037
CAD x time interaction	3.26	0.64	0.000	-0.48	0.32	0.143
HAM x time interaction	0.31	1.07	0.776	-0.24	0.59	0.683
HY x time interaction	0.36	0.98	0.716	-0.02	0.57	0.978
*Diastolic BP*						
CAD	72.70	1.57	0.000	77.65	2.38	0.000
HAM	6.89	2.63	0.013	4.76	4.41	0.289
HY	1.88	2.63	0.480	-6.30	4.34	0.157
CAD x time interaction	1.39	0.49	0.011	0.11	0.15	0.441
HAM x time interaction	-0.57	0.82	0.492	0.07	0.26	0.783
HY x time interaction	-1.36	0.83	0.119	0.53	0.25	0.037

Mixed linear models were performed separately for time periods between baseline and 5 min as well as between 5 min and 14 min after exercise cessation. Shown are treatment contrasts with CAD as reference group. Number of available data for pPWV is n = 18 for CAD, n = 10 for HAM, n = 10 for HY, and for systolic and diastolic BP n = 17 for CAD, n = 7 for HAM, and n = 7 for HY. For pPWV at 5 min data was available for n = 14 in CAD, n = 8 in HAM, and n = 10 in HY.

pPWV, peripheral pulse wave velocity; BP, blood pressure; CAD, coronary artery disease group; HAM, healthy age-matched group; HY, healthy young group.

Systolic BP increased in all groups similarly from baseline to 5 min post-exercise (CAD group: 3.26 mmHg/min, p<0.001) without a linear trend thereafter in any group (Fig 1). Diastolic BP increased by 1.39 mmHg/min in the CAD group (p = 0.011) from baseline to 5 min post-exercise, with insignificantly smaller increases in the other groups (Table 2 and Fig 1). Between min 5 and min 14, diastolic BP remained stable in the CAD and HAM group, but increased significantly in the HY group (by 0.64 mmHg per min, p = 0.037, Table 3 and Fig 1).

Resting HR was comparable between the three groups. Peak HR was significantly lower in the CAD group compared both to HAM (-29.2 bpm, p<0.0001) and HY (-45.0 bpm, p<0.0001). At 5 min post-exercise the CAD group had lower HR compared to HAM (-10.0 bpm, p = 0.057) and HY (-21.4 bpm, p = 0.0001), with a comparable decrease until 14 min post-exercise thereafter (Fig 1). While normal distribution was acceptable for all parameters in all groups at most time points, box plots are shown because of the small group sizes of HAM and HY.

The following variables had Spearman correlation coefficients ≥ 0.3 with delta pPWV: Age (0.48), cPWV at baseline (0.34), and delta diastolic BP (0.32). The mixed linear model that explained the greatest adjusted percentage of variance in delta pPWV was when only age and delta diastolic BP were included (r^2^ = 0.24, p = 0.008).

## Discussion

This is the first study to show that the drop in pPWV found in the legs of YH following a bout of exhaustive exercise was absent in CAD patients and HAM. Similar to previous studies [5,6,26], our YH group showed a decrease in pPWV of 17% at 5 min after peak exercise compared to pre-exercise. Confirming our hypothesis, this drop was absent in the CAD patients, but contrary to our hypothesis, this drop was also absent in HAM, leading to the interpretation that the absence of the drop was not due to an altered physiology with CAD but more likely due to age. Namely, change in pPWV from pre- to post-exercise was related to age, cPWV at rest, and change in diastolic BP, but not to pPWV pre-exercise. Brachial systolic BP was consistently 10 mmHg and diastolic BP 5–8 mmHg lower in CAD patients compared to age-matched healthy controls (significance reached not at all time points).

Most existing studies on post-exercise pPWV have been performed with healthy young volunteers. They have generally found a decrease in pPWV measured at the leg immediately post-exercise (<5 min) persisting further into the recovery period (> 5 min) [26]. To our knowledge, there are no studies who have compared the effect of acute exercise on leg pPWV in older healthy subjects to CAD patients.

Information on pPWV acutely after exercise in middle-aged to elderly persons is sparse, with only few studies who have assessed other parameters of arterial compliance than PWV. Only one study compared middle-aged CAD patients with a control group of patients with chest pain but no CAD [28]. They found post-exercise decreases in ankle-brachial PWV in both groups. However, their measure of PWV was a composite measure of central and peripheral arteries, therefore, comparison with our results of post-exercise pPWV is difficult.

Post-exercise pPWV is likely to reflect endothelial function dependent flow-mediated vasodilation due to exercise-induced hyperemia, resulting in a greater arterial radius, a lesser wall thickness and probably reduced elastic modulus due to the absence of vasoconstriction. A similar vasodilatory response to what is found after exercise can be achieved by occlusion. There is an immediate vasodilation post-exercise lasting up to 20 min probably due to central sympathetic inhibition and local dilatory mechanisms and a sustained post-exercise vasodilation lasting up to several hours due to mainly histamine H1 and H2 receptor activation [29]. We can therefore assume that in our study participants, at 5–15 min post-exercise, peripheral vessels to exercising muscles were dilated, leg blood flow elevated, and vascular smooth muscle tone largely abolished [30].

It is likely that post-exercise pPWV reflects two concomitant vessel characteristics: vessel dilation due to increased shear stress, which theoretically lowers pPWV via an increased vessel diameter, a decrease in vasomotor tone, and a reduced vessel wall thickness. However, in a fully dilated vessel, the passive structures of the vessel wall may be stiffer due to the elastic modulus of the wall being dominated by the collagen fibers rather than the more elastic elastin fibers [31]. This would explain why in older individuals (our CAD and HAM group), despite the most likely dilated arteries, post-exercise pPWV was not reduced. It is likely that, similar to the central arteries, the passive wall structure of their peripheral arteries were stiffer than those of young individuals, but that this higher stiffness was not detected at rest, when the effect of vasomotor tone had an overriding effect on pPWV. Age-associated changes of the brachial, radial and popliteal artery have been found as an increase in lumen diameter and intima media thickness, but not for compliance.[32–34] However, these measurements have been performed at rest and not in the dilated state after acute exercise. Post-exercise, pPWV in the HY may have decreased due to higher elasticity of the passive vessel wall structures of the dilated femoral artery compared to the older subjects. Stiffer arterial intrinsic wall properties due to accumulation of collagens and proteoglycans[35] are likely to be detectable only in the vasodilated state when not masked by the amount of smooth muscle tone, as suggested previously by Naka and colleagues [5]. In fact, we found a significant linear correlation between the change in pPWV from baseline to 5 min post-exercise and age, explaining 25% of the total variance in delta pPWV (p = 0.001). The only other variables that were associated with delta pPWV was resting cPWV (r^2^ = 0.13, p = 0.015) and delta diastolic BP (r^2^ = 0.11, p = 0.064), however, changes in diastolic BP from pre- to post-exercise were similar in all groups.

Surprisingly, in our study cPWV was comparable in CAD patients and age-matched healthy controls. This is in contrast to a study by Hofmann and colleagues [36], who found higher cPWV in CAD patients compared to normal reference values and who found cPWV to increase with age and increasing severity of coronary vessel disease. We did not find an association of cPWV with severity of vessel disease. However, heart rate, systolic and diastolic BP were all reduced in CAD patients compared to our healthy age-matched controls, probably as a consequence of medication, namely all CAD patients were on beta-blockers and 95% on ACE-inhibitors or Sartans. In contrast, in the study cited above [36], systolic BP was higher in CAD patients than controls and was considerably higher than in our CAD patients. Consequently, in our study we can not automatically conclude that central arterial stiffness was comparable in the two groups, since cPWV may have been lowered secondary to the BP-lowering effect of beta-blockers [37]. CPWV has been shown to directly depend on systolic and diastolic BP by approximately 1 m/s for every 10 mmHg change in diastolic BP [20,38–40]. Similarly, pPWV at baseline and after cessation of exercise was comparable between CAD and HAM, however, both systolic and diastolic BP were significantly lower in the CAD compared to the HAM group pre-exercise with a continuing trend to remain lower after exercise (Fig 1), suggesting that peripheral arteries in CAD were either stiffer or their diameter smaller.

### Limitations

PWV is only a surrogate for arterial stiffness and directly dependent on other factors like vessel diameter, intrinsic wall stiffness (including wall thickness and elastic modulus), and blood viscosity, and indirectly dependent on blood pressure [20] and to a lesser extent heart rate [41].

As we did not measure sympathetic nerve activity, we cannot exclude that vasodilation was incomplete post-exercise in our CAD patients and middle-aged healthy subjects.

PWV measurements have known limitations with regard to precision, with reproducibilities of between 5–10% [42,43]. Reproducibility of post-exercise lower limb PVW was performed in five subjects and shown visually but not quantified [5].

We could not measure central and peripheral PWV simultaneously, which prevented us from also measuring cPWV following acute exercise. However, a recent meta-analysis concluded that effects of aerobic exercise on cPWV were equivocal,[14] while they have been well established for pPWV.

Further, we did not measure blood viscosity and can therefore not exclude the group difference to be due to different responses in blood viscosity. Namely, exercise has been shown to increase blood viscosity immediately post-exercise by approximately 12%-20% in young healthy volunteers [5,44,45].

Last but not least, the sample sizes of the healthy groups was small, suggesting that the distinction between the CAD and HAM group may have lacked statistical power. However, the differences in SBP and DBP between CAD and HAM group pre-exercise were significant, supporting that the absence of a difference between these two groups in pPWV and cPWV pre-exercise was likely due to a lower BP of the CAD group and did not imply comparable arterial stiffness.

## Conclusions

We conclude that post-exercise dilatation of the leg arteries did not lead to a concomitant decrease in pPWV in CAD patients and age-matched healthy controls, as was found in our young healthy population. We suggest that the reason for this may be a stiffer vessel wall of the dilated arteries in older subjects, as the change in pPWV was related to age and cPWV at rest. Post-exercise measurements may offer a maneuver to assess intrinsic stiffness of peripheral arteries. It remains to be studied in longitudinal intervention studies whether this parameter can be influenced by chronic exercise training, however, the absence of a difference between our CAD and HAM group does not make this probable.

## Supporting information

S1 File S1_File. (XLSX)
